# Supplementary material for: Construction of Magnetic Composite Bacterial Carrier and Application in 17β-Estradiol Degradation
Source: Molecules. 2022 Sep 7;27(18):5807. doi: 10.3390/molecules27185807 (PMC9504236; doi:10.3390/molecules27185807)
Supplement: Supplementary file 1 [file molecules-27-05807-s001.zip › molecules-1885239-supplementary.pdf]

Supplementary Data

Construction of Magnetic Composite Bacterial Carrier and Application in 17*β*-estradiol Degradation

Molecules

Sicheng Wu[1], Peng Hao[1], Zongshuo Lv[1], Xiqing Zhang[1], Lixia Wang[2] Wangdui Basang[3], Yanbin Zhu[3] & Yunhang Gao[1,*]

[1]College of Animal Science and Technology, Jilin Agricultural University, Changchun, Jilin 130118, China

[2]Northeast Institute of Geography and Agroecology, Chinese Academy of Sciences, Changchun, Jilin 130102, China

[3]Institute of Animal Husbandry and Veterinary Medicine, Tibet Academy of Agricultural and Animal Husbandry Science, Lhasa, 850009, China

*Corresponding author: Yunhang Gao; e-mail: gaoyunhang@163.com; phone: +86-13159752912;

Supplementary Tables

Table S1: Shape of microspheres at different ratios

P+S	P:S
2%	9:1
	7:3
	5:5
	3:7
	1:9
4%	9:1
	7:3
	5:5
	3:7
	1:9
6%	9:1
	7:3
	5:5
	3:7
	1:9
8%	9:1
	7:3

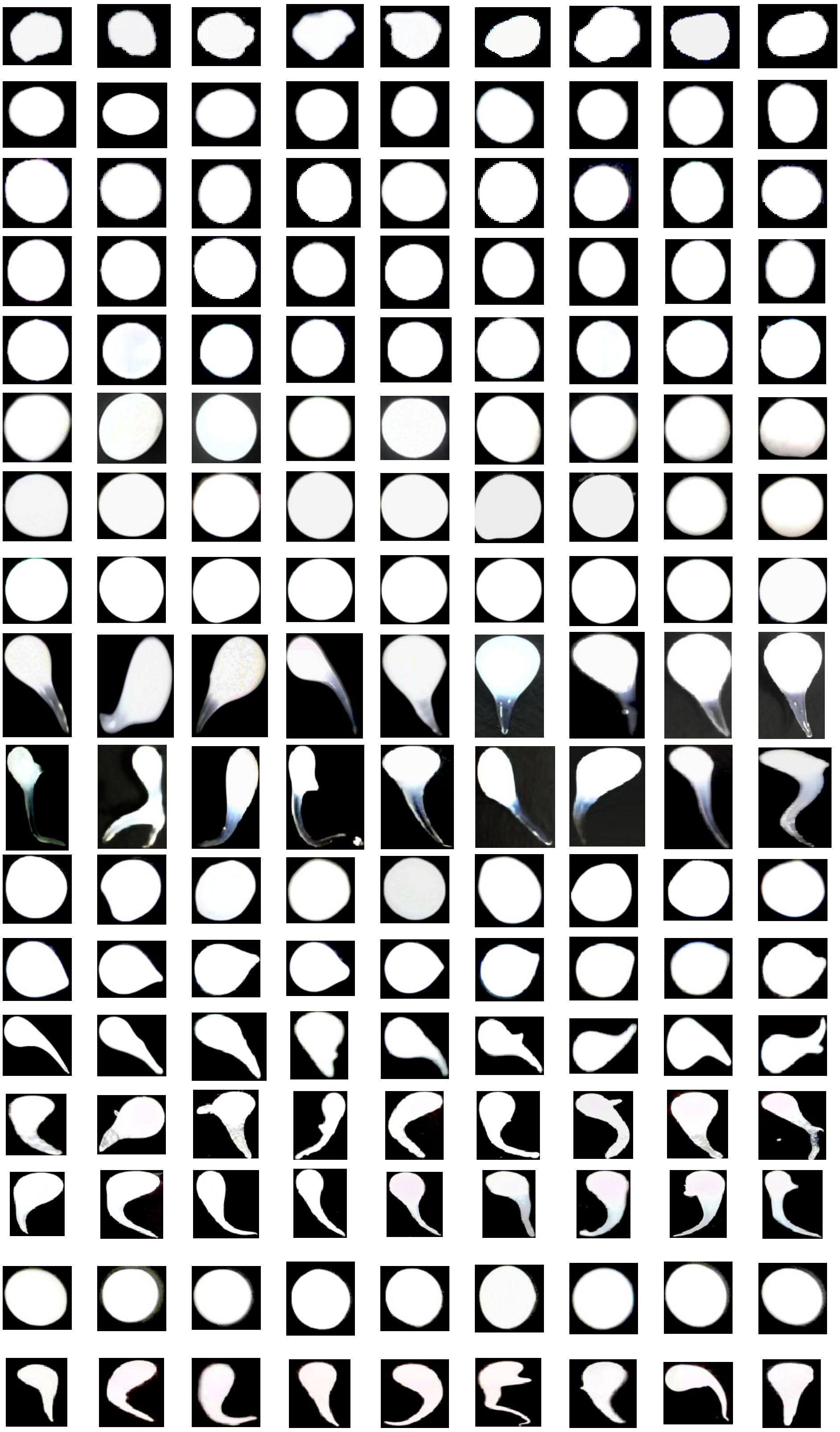

	5:5	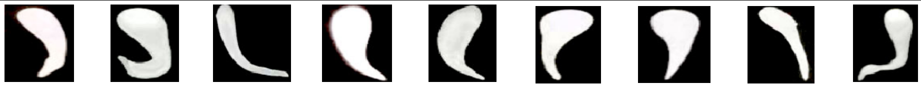
	3:7	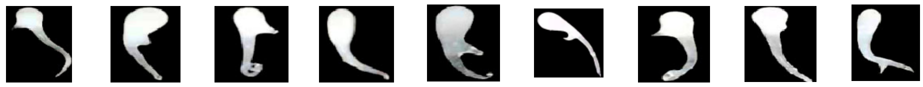
	1:9	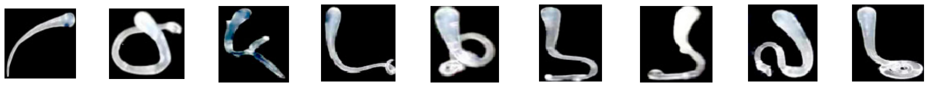
	9:1	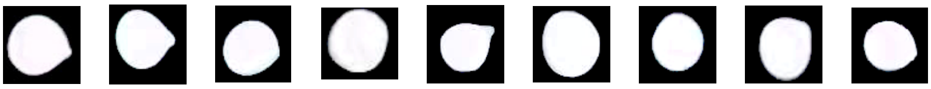
	7:3	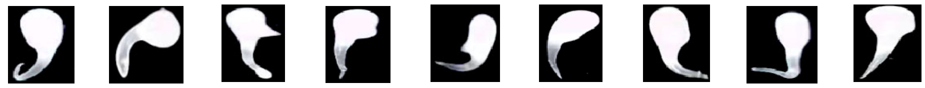
10%	5:5	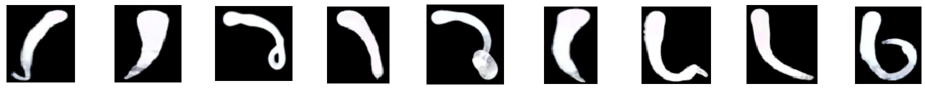
	3:7	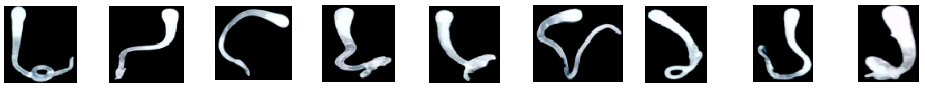
	1:9	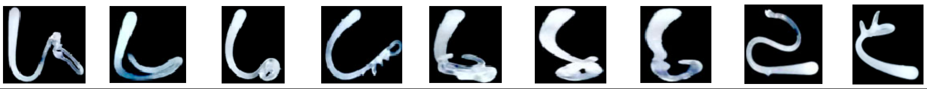

PVA: polyvinyl alcohol

SA: sodium alginate

Figure S1

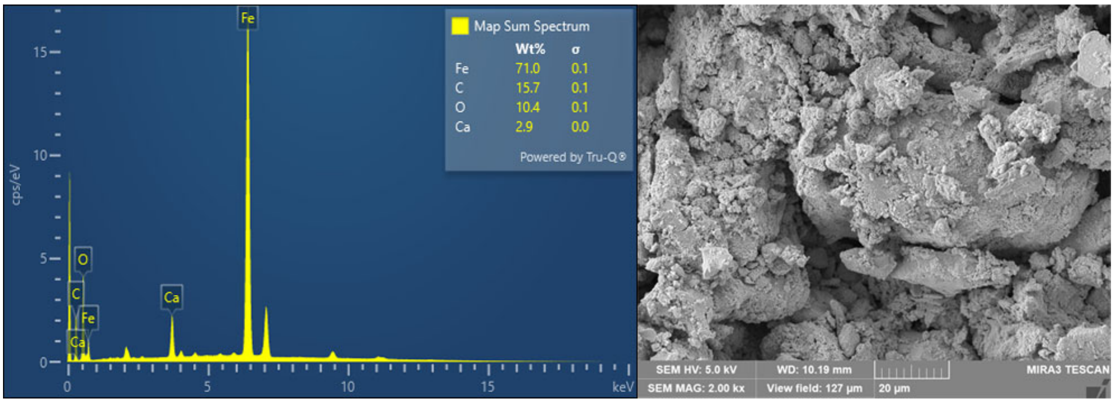

Figure S1: The chemical composition and the SEM image of composite carrier internal structure (2000x); scale bar is 20 μm
